# Pan-cancer clinicopathological and genomic characteristics of peritoneal metastasis

**DOI:** 10.1038/s41698-025-01227-7

**Published:** 2025-12-13

**Authors:** Tianwei Chen, Yebin Yang, Xiaoli Liu, Fanhe Dong, Liang Wang, Yuqiang Shan, Xiang Wang

**Affiliations:** 1 Zhejiang Key Laboratory of Zero Magnetic Medicine, Affiliated Hangzhou First People’s Hospital, School of Medicine, Westlake University, Hangzhou, China; 2 Department of Gastrointestinal Surgery, Affiliated Hangzhou First People’s Hospital, School of Medicine, Westlake University, Hangzhou, China; 3 The Fourth School of Clinical Medicine, Zhejiang Chinese Medical University, Hangzhou First People’s Hospital, Hangzhou, China

**Keywords:** Biomarkers, Cancer, Computational biology and bioinformatics, Genetics, Oncology

## Abstract

Peritoneal metastasis (PM) poses a significant clinical challenge, yet the patient characteristics and genomic drivers underlying PM remain poorly characterized. Leveraging the MSK-MetTropism cohort (*n* = 25,755), our pan-cancer analysis reveals PM occurs in 29% of metastatic patients with extreme incidence variation (1% in THPA to 92% in HGSOC), and digestive/gynecologic malignancies exhibit the highest PM propensity with significant sex disparities. PM confers worse survival in 11/39 subtypes. Genomic profiling of 5,942 PM patients identifies enriched mutations in *ESR1*, *TCF7L2*, and FBXW7, pathway-level of TGF-Beta mutation, and subtype-specific drivers, including *RET* mutations in gastric PM. Mutational signatures implicate ROS (SBS18), HR deficiency (SBS3), and SBS8 across ≥9 cancer types. These results establish foundational insights into PM biology, though future PM tissue profiling is warranted to overcome primary tumor bias in genomic data.

## Introduction

Peritoneal metastasis (PM) represents an advanced-stage manifestation of cancer. Epidemiological studies indicate that PM primarily originates from gastrointestinal (GI), reproductive, and genitourinary tract cancers, accounting for approximately 90% of diagnosed PM cases^1^. PM is less frequently observed in patients with breast cancer, lung cancer, and malignant melanoma^2^. Compared to other metastatic sites, diagnosing PM is challenging due to the lower accuracy of conventional imaging techniques^3^. Furthermore, treatment options for PM are limited, as it often exhibits resistance to systemic therapy, consequently, regional chemotherapy is routinely employed^4^. Patients with PM typically experience poor prognoses in gastric^5^, colorectal^6^, pancreatic^7^, and female reproductive cancers^8^. However, the incidence and patient profiles of PM across a pan-cancer perspective remain relatively unexplored.

Genomic characteristics of site-specific PM from different cancer subtypes are occasionally reported. Studies have shown higher PM prevalence in gastric cancer patients harboring *SOX9* and *DEK* mutations^9^, colorectal cancer patients with KRAS/BRAF mutations^10^, and appendiceal cancer patients with GNAS mutations^11^. Genomic alterations in PM from other cancer types are less frequently documented, likely due to the lower incidence of PM in these malignancies. The resistance of PM to systemic chemotherapy is attributed, in part, to the peritoneal-plasma barrier^12^. Regional therapies, specifically cytoreductive surgery (CRS) combined with hyperthermic intraperitoneal chemotherapy (HIPEC), represent the more established treatment modalities for PM^13^, although the efficacy of HIPEC remains controversial^14^. Elucidating the genomic alterations underlying PM holds significant potential for understanding its pathogenesis and developing novel diagnostic and therapeutic strategies.

Leveraging real-world statistics and genomic data from the MSK-MetTropism cohort—a large dataset comprising >25,000 metastatic cancer patients—we analyzed the prevalence of peritoneal metastasis (PM) across various cancer types and characterized the profiles of PM patients. Additionally, we investigated mutational hotspots and mutational signatures from a pan-cancer perspective. These findings significantly contribute to a deeper understanding of the patient characteristics and genomic landscape associated with PM.

## Results

### Overview of PM patients in metastatic cancer patients

**Fig. 1:**
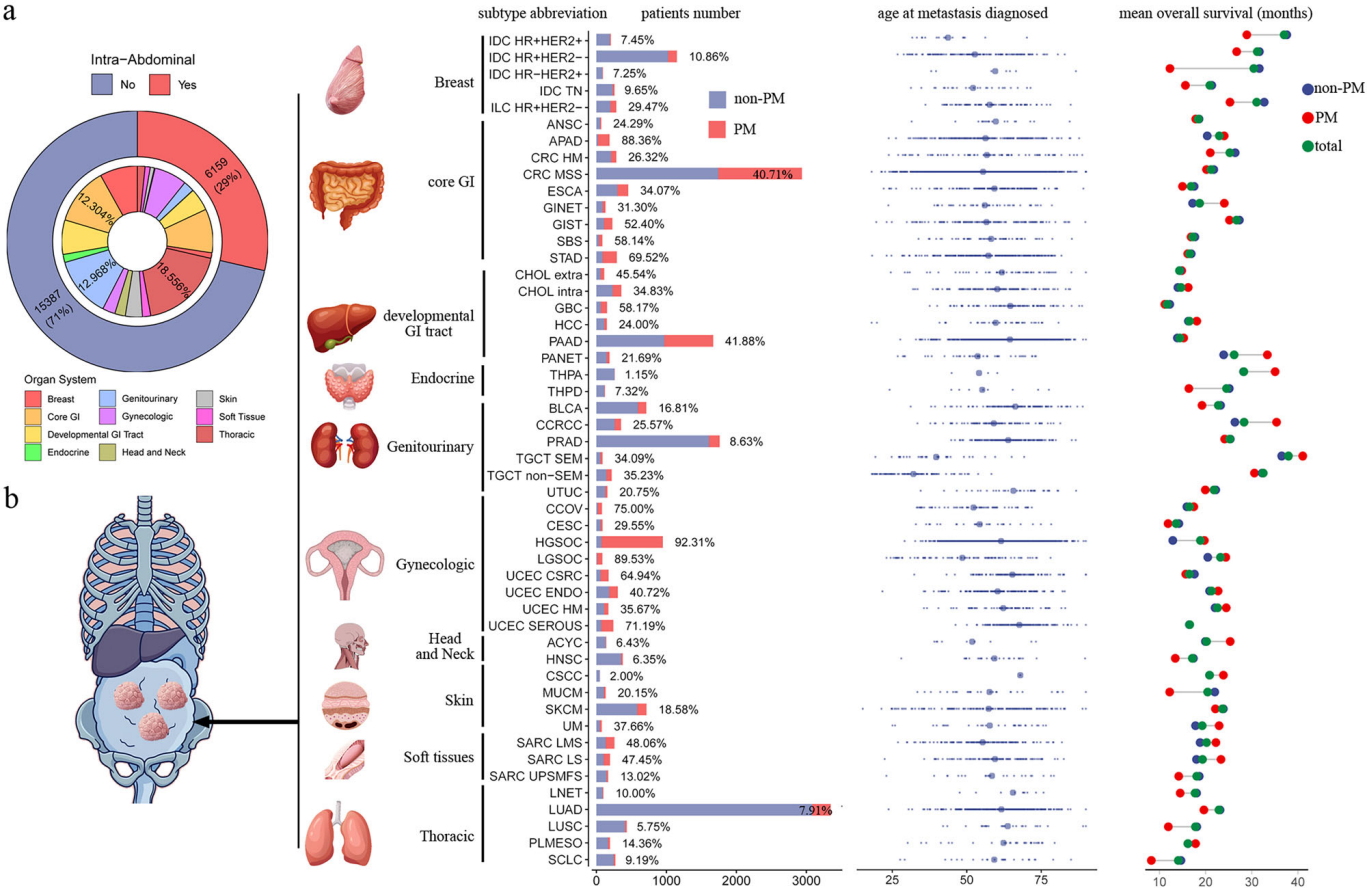
Overview of PM patients in this cohort. **a** Proportions of intra-abdominal metastases by involved organ system. **b** Integrating anatomical mapping with quantitative features across cancer subtypes. The schematic shows metastatic sites in the abdomen, with bar and dot plots detailing subtype-specific characteristics.

The Memorial Sloan Kettering-Integrated Mutation Profiling of Actionable Cancer Targets (MSK-IMPACT) MetTropism program provided the foundation for this study. This cohort comprises 25,755 metastatic patients spanning 50 cancer subtypes across 10 organ systems, with detailed records of metastatic sites. Patients were designated as having peritoneal metastasis (PM) if they exhibited one or more metastatic lesions within the peritoneum. Within this cohort, we identified 6159 patients with PM, representing 29% of all metastatic patients (Fig. 1a). Consistent with prior reports, the proportion of metastatic patients developing PM varied substantially across cancer subtypes, ranging from 92% in high-grade serous ovarian carcinoma (HGSOC) to 1.1% in thyroid papillary carcinoma (THPA) (Fig. 1b). Notably, the PM proportion also varied significantly among cancer subtypes originating from the same organ system, potentially reflecting differences in their developmental biology. For example, within cancers of the digestive tract [core gastrointestinal (GI)], PM accounted for 88% of metastatic appendiceal adenocarcinoma (APAD) and 69% of metastatic stomach adenocarcinoma (STAD), while the PM proportion was substantially lower in anal squamous cell carcinoma (ANSC) (24%) and hypermutated colorectal cancer (HM CRC) (26%). Furthermore, PM prevalence was notably high in cancers originating from gynecologic organs, the core GI tract, and the developmentally derived GI tract, consistent with prior reports. Among the 6159 PM patients, the age at first metastasis diagnosis was typically >50 years. Notable exceptions included testicular non-seminoma (TCGT non-SEM; mean age 32), testicular seminoma (TCGT SEM; mean age 39), hormone receptor-positive HER2-positive invasive ductal breast carcinoma (IDC HR + HER2+; mean age 43), and low-grade serous ovarian carcinoma (LGSOC; mean age 48) (Fig. 1b). Moreover, we summarized the synchronous metastatic sites in patients with PM. The most common sites were the liver (63%), lung (39%), and bowel (39%), with detailed dissemination patterns provided in Supplementary Fig 1a and Supplementary Data 1. The average survival time for PM patients varied significantly by primary tumor type, ranging from very short in small-cell lung cancer (SCLC; 8.2 months) to considerably longer in testicular germ cell tumors (TGCT; 41 months) and clear cell renal cell carcinoma (CCRCC; 35 months) (Fig. 1b). Intriguingly, patients with PM exhibited longer survival compared to those with other metastatic sites in certain cancers, such as pancreatic neuroendocrine tumors (PANET) and CCRCC. Conversely, PM was associated with shorter mean overall survival in breast and lung cancers (Fig. 1b).

### Patient characteristics of PM patients

**Fig. 2:**
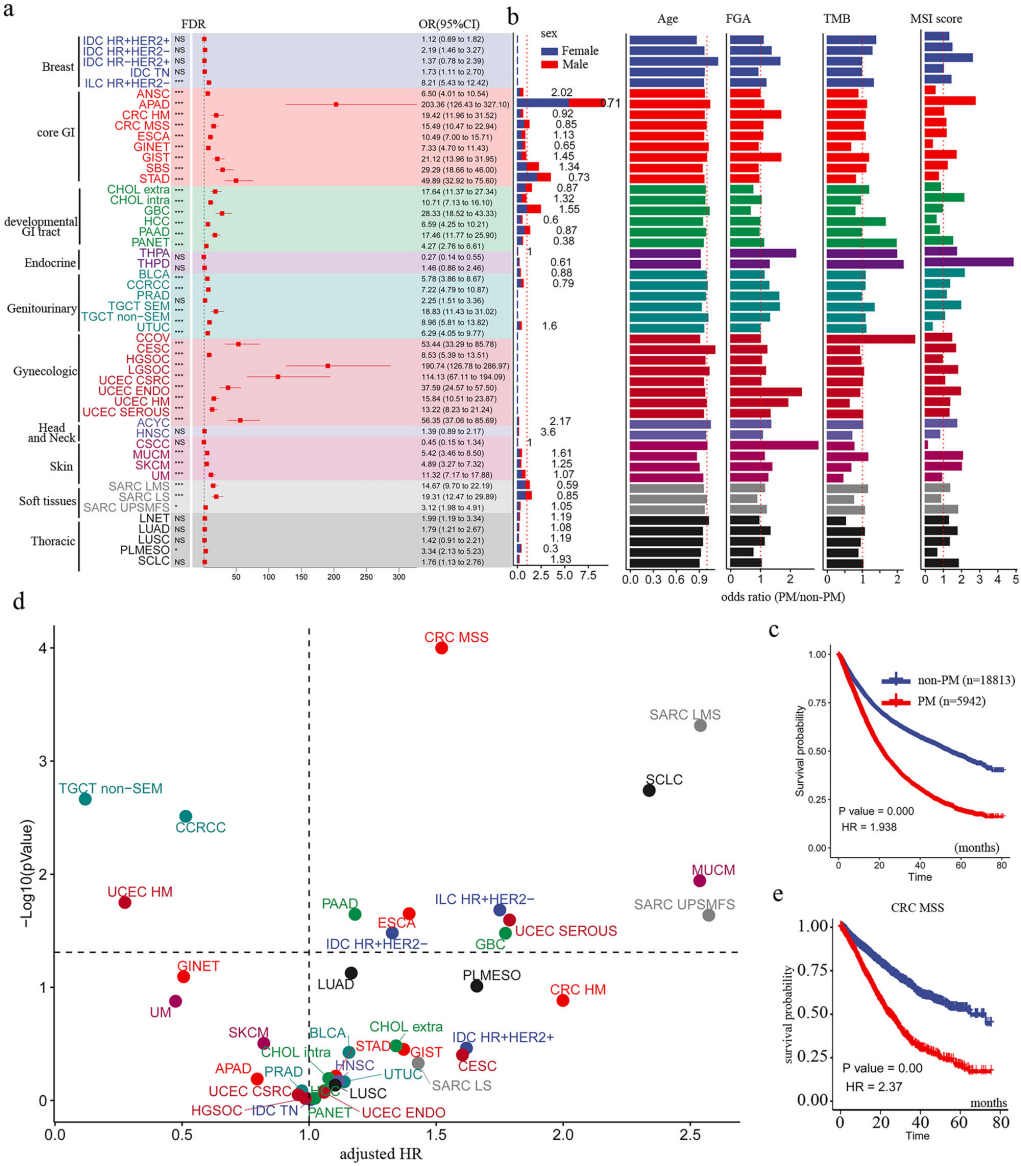
Patients’ Characteristics and Prognostic Analysis of PM. **a** Forest plot displays odds ratios for peritoneal metastasis (PM) occurrence across cancer types. Asterisks indicate statistical significance: *FDR < 0.05, ***FDR < 0.001. **b** Distribution of key genomic features: fraction of genome altered (FGA), tumor mutational burden (TMB), and microsatellite instability (MSI). **c** Kaplan–Meier analysis of overall survival between PM (*n* = 5942) and non-PM (*n* = 18,813) patients (HR = 1.938, *P* < 0.001). **d** Scatter plot of adjusted hazard ratios for PM risk across cancer types. *X*-axis: adjusted HR; *y*-axis: −log₁₀(*P*-value); dot color indicates cancer type; dashed line: *P* = 0.05 threshold. **e** Subgroup survival analysis in colorectal cancer (microsatellite-stable subtype) shows significantly worse prognosis in PM cases (HR = 2.37, *P* < 0.001).

To comprehensively evaluate PM occurrence across 50 cancer types, we calculated odds ratios (ORs) by training a multivariable logistic model with explanatory variables including cancer subtype, gene panel, sex, age, etc. (see methods). Model accuracy reached 0.82 and 0.79 for the train and test datasets, respectively. To this end, cancers originating from breast, thoracic, head and neck, and endocrine organs exhibited significantly lower odds of peritoneal metastasis (Fig. 2a**;** Supplementary Data 2). Conversely, cancers from the core GI tract (excluding anal squamous cell carcinoma [ANSC] and hypermutated colorectal cancer [HM CRC]), developmentally derived GI tract (excluding hepatocellular carcinoma [HCC] and pancreatic neuroendocrine tumors [PANET]), gynecologic organs (excluding endometrioid endometrial carcinoma [UCEC ENDO] and hypermutated endometrial carcinoma [UCEC HM]), and soft tissues (excluding undifferentiated pleomorphic sarcoma/malignant fibrous histiocytoma [UPSMFS]) demonstrated significantly higher odds of PM (Fig. 2a). Strikingly, high-grade serous ovarian carcinoma [HGSOC; OR(95%CI) = 190.74(126.78–286.97), FDR < 0.001], low-grade serous ovarian carcinoma [LGSOC; OR(95%CI) = 114.13(67.11–194.09), FDR < 0.001], and appendiceal adenocarcinoma [APAD; OR(95%CI) = 203.36(126.43–327.10), FDR < 0.001] showed substantially elevated ORs compared to other cancers, indicating markedly increased peritoneal metastatic risk. We also observed significant sex disparities: males had higher PM frequency in ANSC, gallbladder carcinoma (GBC), head and neck squamous cell carcinoma (HNSC), and small-cell lung cancer (SCLC), while females showed higher PM frequency in gastrointestinal neuroendocrine tumors (GINET), stomach adenocarcinoma (STAD), HCC, PANET, thyroid papillary carcinoma (THPD), clear cell renal cell carcinoma (CCRCC), leiomyosarcoma (SARC LMS), and pleural mesothelioma (PLMESO) (Fig. 2b). Regarding molecular characteristics, most PM tumors exhibited higher chromosomal instability as measured by the fraction of genome altered (FGA) (e.g., cervical squamous cell carcinoma [CSCC], HM CRC), excepting extrahepatic cholangiocarcinoma (CHOL extra), GBC, and PLMESO. Tumor mutation burden (TMB), a biomarker for immunotherapy, was generally similar in PM and primary tumors across most subtypes; however, PM in GINET, STAD, GBC, UCEC HM, HNSC, and skin cancers demonstrated lower TMB. While most PM subtypes had high microsatellite instability (MSI) scores, GINET, HCC, upper tract urothelial carcinoma (UTUC), CSCC, and PLMESO PM exhibited low MSI (Fig. 2b). Survival analysis revealed that PM patients (*n* = 5942) had significantly shorter survival than those with other metastatic sites (HR = 1.93, *P* < 0.001; Fig. 2c), indicating poorer prognosis. Multivariable Cox regression analysis of 39 cancer subtypes (requiring >10 PM cases and >100 total cases) confirmed significantly worse survival in PM groups for 11 subtypes (Fig. 2d, Supplementary Data 3). Notably, while HGSOC and APAD had high PM incidence, survival was unaffected by PM status; conversely, CRC MSS exhibited both high PM occurrence and PM-associated survival detriment (HR = 2.37, *P* < 0.001; Fig. 2E). Soft tissue sarcomas (SARC LMS and SARC UPSMFS) and lung cancer subtypes (SCLC) conferred poor prognosis upon PM occurrence (Fig. 2d**;** Supplementary Fig 1b, c), whereas liver cancers [HCC, cholangiocarcinoma (CHOL)] showed no significant survival difference with PM (Fig. 2d**;** Supplementary Fig 1d).

### Gene mutation profile of PM

**Fig. 3:**
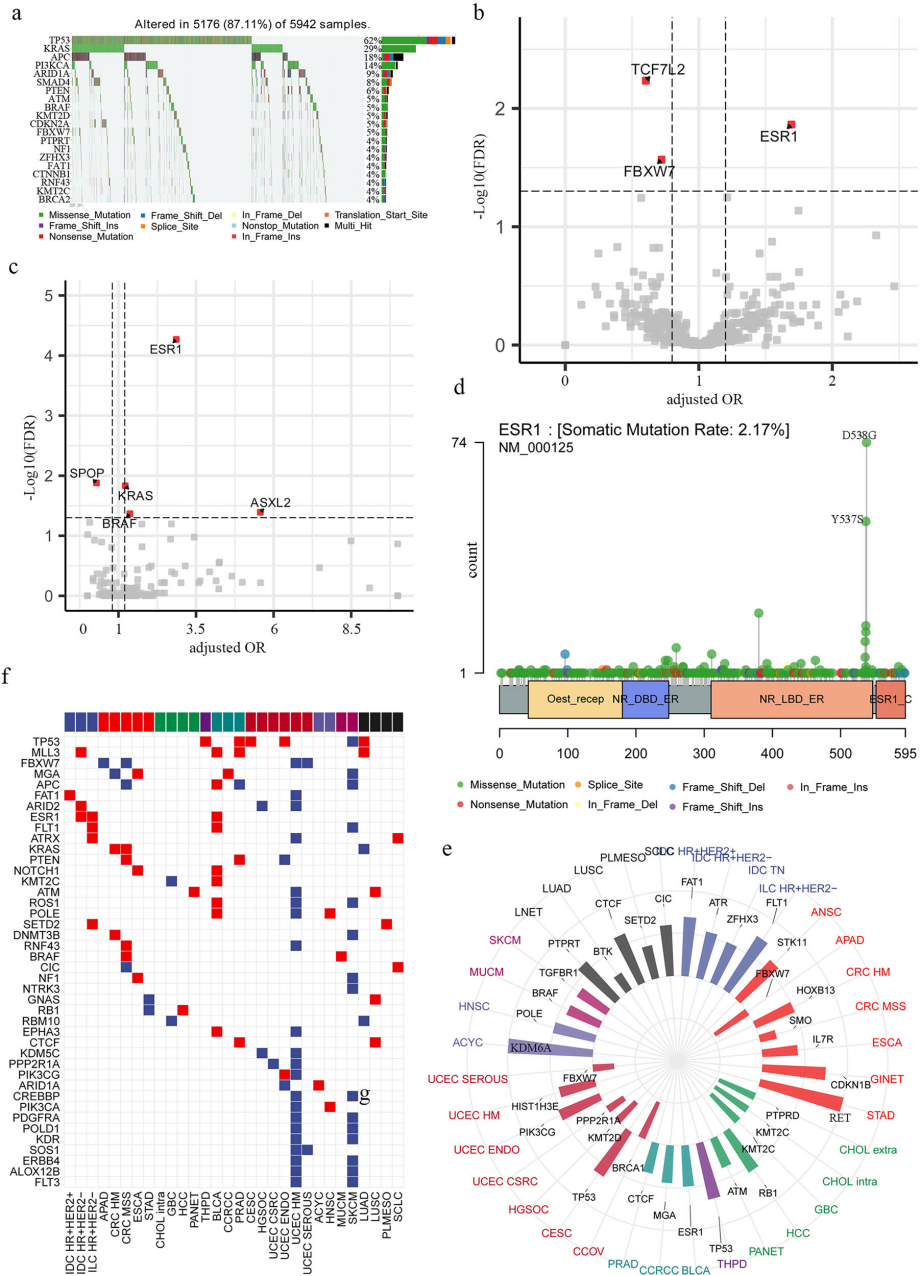
Genomic alterations of PM patients. **a** Waterfall plot of recurrently mutated hotspot genes in patients with peritoneal metastasis (PM). **b** bVolcano plot identifying genes with significantly altered mutation frequencies in PM compared to non-PM samples. **c** Volcano plot of genes harboring hotspot mutations that are significantly enriched or depleted in PM versus non-PM samples. **d** Lollipop plot of the ESR1 mutation spectrum across the MetTropism cohort, with annotated recurrent hotspot residues. **e** Circos plot summarizing the distribution of genomic alterations (mutations and copy-number changes) across cancer types; radial bars represent alteration frequencies of selected genes in each subtype. **f** Heatmap of alteration status for selected genes across cancer subtypes, with red indicating enrichment and blue indicating depletion in PM.

Among 6159 PM patients, 5942 had targeted sequencing data (Gene panel: IMPACT341: *n* = 490; IMPACT410: *n* = 1584; IMPACT468: *n* = 3868). Key hotspot mutations in PM versus non-PM cohorts included TP53 (62% vs 47%, OR = 1.83, FDR < 0.001), KRAS (29% vs 20%, OR = 1.60, FDR < 0.001), and APC (18% vs 15%, OR = 1.42, FDR < 0.001), with SMAD4 mutation being notably enriched (8% vs 5%, OR = 1.85, FDR < 0.001; Fig. 3a, Supplementary Fig 2a). Comprehensive OR analysis with multivariable logistic regression analysis, adjusted with cancer subtype, gene panel et al. (see method), of PM versus non-PM revealed significantly enriched mutations in ESR1 [OR(95%CI) = 1.70(1.45–1.98), FDR = 0.01], TCF7L2 [OR(95%CI) = 0.60(0.52–0.69), FDR = 0.006], FBXW7[OR(95CI) = 0.72(0.65–0.80), FDR = 0.03] (Fig. 3b**;** Supplementary Data 4). It is intriguing to identify ESR1 mutation as a positive regulator in peritoneal metastasis (PM) formation. While ESR1 mutation has previously been reported to promote breast cancer metastasis to gynecological organs^15^, its role in PM formation remains unreported. To determine whether genetic mutations in ESR1 and TCF7L2 influence their transcriptional activity, we applied a protein–protein interaction (PPI)-based deconvolution approach to two cohorts with matched mutation and RNA-seq data: a metastatic breast cancer cohort (*n* = 379)^16^ and TCGA-COAD (GDC dataset). The analysis revealed that tumors harboring ESR1 mutations exhibited significantly elevated transcriptional activity of ESR1 (Supplementary Fig. 2b), while colorectal tumors carrying TCF7L2 mutations showed decreased transcriptional activity of TCF7L2 (Supplementary Fig. 2c). To further investigate hotspot cancer mutations involved in PM, we analyzed 1126 hotspot mutation sites across 240 genes^17^. Through this approach, we identified 5 genes with hotspot mutations affecting PM: ESR1 [OR(95%CI) = 2.86(2.28–3.59), FDR < 0.001], SPOP [OR(95%CI) = 0.29(0.20–0.43), FDR = 0.013], KRAS [OR(95%CI) = 1.22(1.14–1.30), FDR = 0.002], ASXL2 [OR(95%CI) = 5.57(2.97–10.45), FDR = 0.041], BRAF [OR(95%CI) = 1.37(1.22–1.54), FDR = 0.043] (Fig. 3c**;** Supplementary Data 5). Moreover, hotspot mutations in ESR1 were predominantly located at amino acid positions Y537 and D538 (Fig. 3d). Building on previous studies demonstrating that these mutations lead to constitutive activation of ESR1 and promote breast cancer metastasis^15^—a finding consistent with our own results—we further investigated whether such hotspot mutations influence gene expression. Here within the metastatic breast cancer cohort as described above^16^ we observed that tumors carrying these hotspot mutations in ESR1 exhibited significantly elevated transcriptional activity of ESR1 (Supplementary Fig. 2b). To more comprehensively assess genomic alterations associated with peritoneal metastasis (PM), we integrated both somatic mutation and copy number alteration (CNA) data. We first confirmed that mutation and CNA events were largely independent at the gene level (*R* = –0.034; Supplementary Fig. 2d). A multivariable logistic regression model adjusted for cancer subtype, gene panel, and sex identified several genes significantly associated with PM risk, including SMAD4 [OR(95%CI) = 1.40 (1.29–1.52), FDR = 0.001], KRAS [OR(95%CI) = 1.29(1.21–1.38), FDR = 0.002], and PAX7 [OR(95%CI) = 0.54(0.44–0.66), FDR = 0.030], in addition to previously reported genes such as ESR1, TCF7L2, and FBXW7 (Supplementary Fig. 2e, Supplementary Data 6). Therefore, our pan-cancer genomic analysis demonstrates a positive correlation between ESR1 hotspot mutations and PM.

**Fig. 4:**
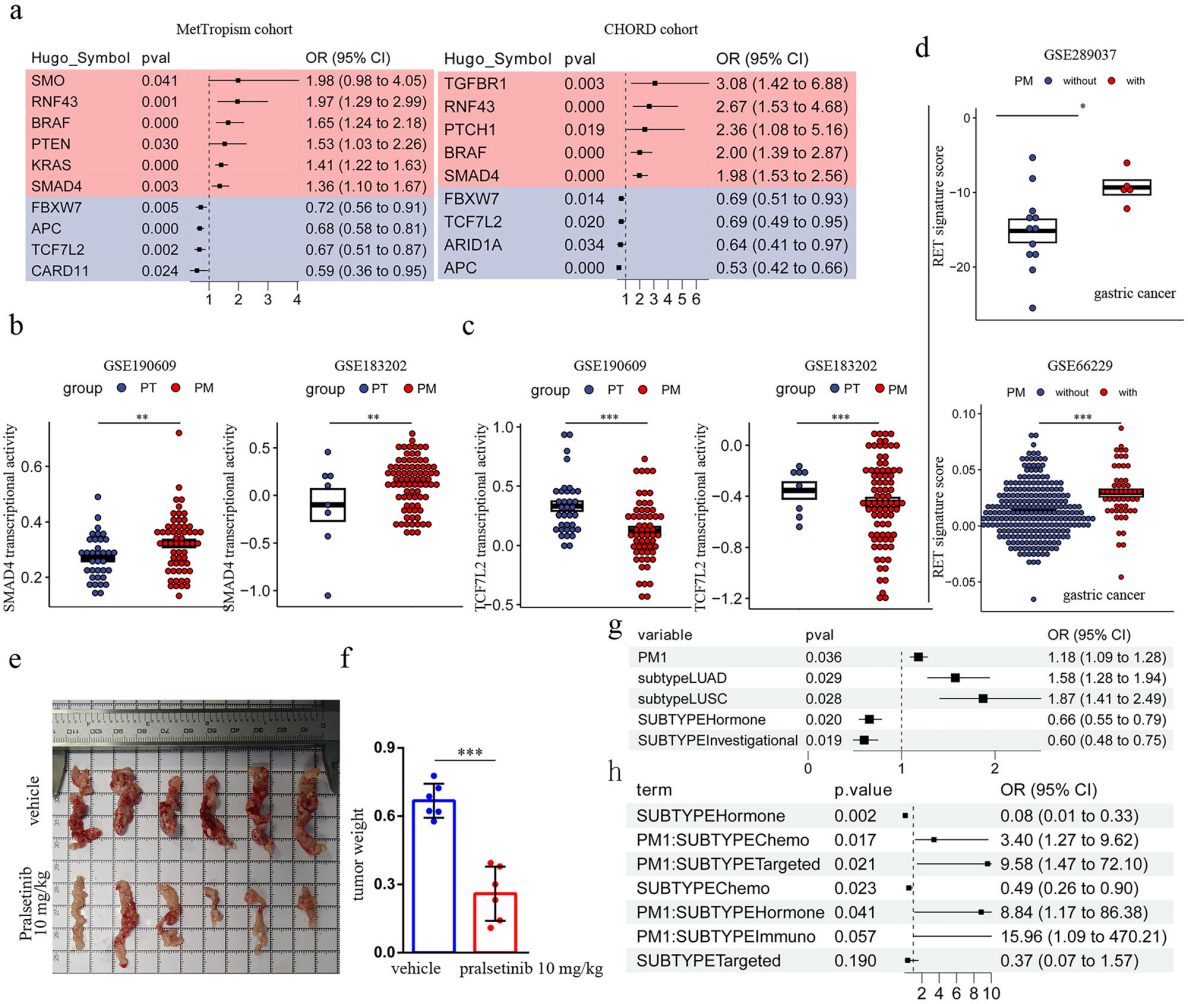
Functional validation of key genomic findings in peritoneal metastasis. **a** Forest plot of gene-level odds ratios for peritoneal metastasis across two CRC cohorts. **b**, **c** Transcriptional activities of SMAD4 (**b**) and TCF7L2 (**c**) inferred from bulk RNA-seq data in two independent colorectal cancer cohorts. PT, primary tumor; PM, peritoneal metastasis. **P < 0.01, ***P < 0.001. **d** RET signature score inferred from bulk RNA-seq data in two independent gastric cancer cohorts. ***P < 0.001. **e** Representative bright-field images of tumors from control and pralsetinib-treated mice. **f** Comparison of tumor weight between control and pralsetinib-treated groups. Data are presented as mean ± SEM; statistical significance was determined by a two-tailed Student’s *t* test. ***P < 0.001. **g**, **h** Multivariable analysis of factors associated with tumor progression. Forest plot displays odds ratios for peritoneal metastasis (PM), cancer subtypes, and treatment modalities from a logistic regression model (**g**) or a logistic regression model that incorporated interaction terms between key variables (**h**).

Subtype-specific risk gene analysis across 50 cancer types identified significant risk/protective genes in 36 subtypes (Supplementary Data 7). Clinically relevant findings included: BRAF [OR(95%CI) = 1.65(1.24–2.18), FDR = 0.04] and KRAS [OR(95%CI) = 1.41(1.22–1.63), FDR = 0.001] mutations in CRC MSS, which were both previously reported; FBXW7 mutations suppressing PM in APAD and UCEC SEROUS despite its pro-metastatic role in other cancers; and CTCF mutations promoting PM in PRAD and LUSC (Fig. 3e). RET mutation (*P* = 0.05, FDR = 0.30), although it was not statistically significant after multiple testing correction (FDR), was the top ranked PM risk factor in STAD (indicating therapeutic potential for RET inhibitors), where all 7 RET mutated patients developed PM. Among the 141 PM-risk/protective genes, 42 recurred across cancers (Fig. 3f). Notable patterns included: TP53 mutations conferring PM risk in THPD/PRAD/CESC/LUAD but protection in SKCM; MLL3 mutations promoting PM in IDC HR + HER2-/BLCA/PRAD/LUAD via enhanced EMT; and contradictory FBXW7 effects requiring further validation. Additional risk genes included ESR1 mutations promoting PM in IDC HR + HER2-, ILC HR + HER2-, and BLCA (Fig. 3f). We next sought to corroborate this finding through an independent analysis of the CHORD dataset^18^. As both datasets originate from MSK and have overlapping patients, we first established a validation cohort of 11,240 patients unique to CHORD (including 2284 PM cases, 20.3%) to prevent bias. In contrast to the MetTropism dataset, which covers 50 cancer subtypes, this CHORD-specific cohort comprised only five: breast cancer (2295), colorectal cancer (CRC, 2412), lung cancer (3252), pancreatic cancer (1406), and prostate cancer (1175). Given the clinical significance of PM in CRC, we focused our validation on mutations enriched or depleted in this cancer type. As a result, we successfully validated 6 out of 10 significant genes in the CHORD dataset (Fig. 4a), thereby corroborating our initial findings. Although the SMO mutation was not significant in CHORD, we identified its direct regulator, PTCH1. Furthermore, since both the TGFβ/SMAD4 and APC/TCF7L2 pathways are frequently implicated in CRC-related PM, we integrated RNA sequencing data to further examine these pathways. In both the GSE190609 and GSE183202 cohorts, SMAD4 transcriptional activity was significantly upregulated in PM tissues compared to primary tumors (Fig. 4b). Conversely, TCF7L2 transcriptional activity was consistently reduced in PM across both cohorts (Fig. 4c). To extend our validation to other cancer types, we focused on gastric cancer (GC), where PM poses the most challenging clinical scenario. We first asked whether the RET signaling pathway is altered in GC patients with PM. Our analysis revealed that the RET pathway activity was significantly elevated in primary GC tumors from patients with PM compared to those without in both GSE289037 and GSE66229 cohorts (Fig. 4d). To evaluate the clinical potential of our findings, we established a gastric PM model using the MFC mouse gastric cancer cell line and treated the mice with intraperitoneal injections of pralsetinib, a clinically used RET inhibitor, every three days. Notably, pralsetinib treatment significantly suppressed PM formation in this model (Fig. 4d, f). This result prompted us to further investigate whether the presence of PM would interfere with cancer therapies. To this end, we identified 3,078 patients (842 with PM) from the MetTropism cohort and 1770 patients (415 with PM) from the CHORD cohort with available therapy information. By building a multivariable logistic regression model, we found that the presence of PM [OR(95%CI) = 1.18 (1.09–1.28), *P* = 0.036] predicted tumor progression (Fig. 4g). To further dissect the association between specific treatment modalities and PM status, we performed a logistic regression analysis incorporating interaction terms in the colorectal cancer (CRC) subgroup. Seven treatment categories were included: Biologic (*n* = 75), Bone Treatment (*n* = 30), Chemo (*n* = 1173), Hormone (*n* = 37), Immuno (*n* = 12), Targeted (*n* = 29), and Investigational (*n* = 14). This analysis revealed that PM patients exhibited significantly higher rates of tumor progression in therapies of Targeted [OR (95% CI) = 9.58 (1.47–72.10), *P* = 0.021], Hormone [OR (95% CI) = 8.84 (1.17–86.38), P = 0.041] and a non-significant increase of Immuno [OR (95% CI) = 15.96 (1.09–470.21), *P* = 0.057], and it is particularly noteworthy that a significant association was also observed in the chemotherapy group [OR (95% CI) = 3.40 (1.27–9.62), P = 0.017] (Fig. 4h), a finding consistent with prior reports indicating that CRC patients with peritoneal metastasis exhibit resistance to systemic chemotherapy^19^. Collectively, our key findings were validated across multiple dimensions of evidence, including genetic, transcriptional, and functional analyses.

### Pathway mutation profile of PM

**Fig. 5:**
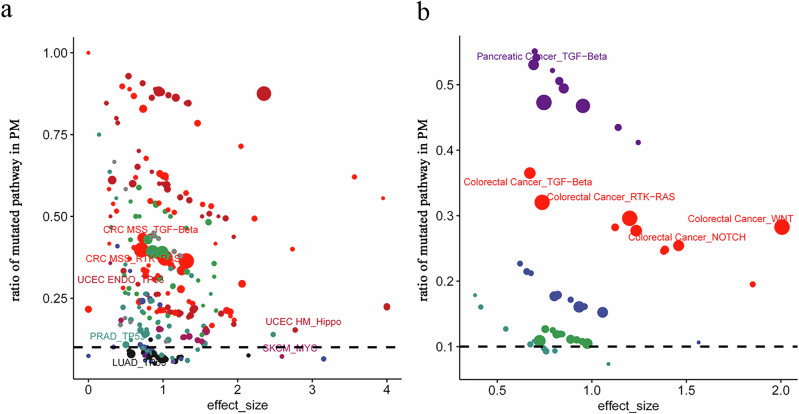
Somatic mutations in key pathways of PM. **a**, **b** Scatter plot comparing effect size of pathway mutation between peritoneal metastasis (PM) and non-PM samples in the (**a**) MetTropism and (**b**) CHORD cohorts. Each point represents a specific pathway, positioned by its effect size (*x*-axis) and the proportion of PM samples carrying the mutation (*y*-axis). A 10% mutation frequency threshold is indicated by a dashed horizontal line. Point color corresponds to cancer subtype, and point size reflects the number of PM samples with the mutated pathway.

We next characterized associations between pathway mutation and PM formation using 334 genes spanning 10 canonical pathways (Supplementary Data 8)^20^. Across most cancer subtypes, PM patients exhibited dramatically reduced mutation rates in cell-cycle pathway genes compared to non-PM cohorts (hedges’s G = 0.83, FDR < 0.01; Supplementary Data 9), except in certain gynecologic cancers (CCOV, HGSOC; Supplementary Fig 3a). Although elevated pathway mutation fractions were observed in the NRF2, PI3K, RTK-RAS, TGF-Beta, and TP53 pathways compared to others, none of the ten pathways showed statistically significant sample-level mutation frequencies (Supplementary Fig 3a**;** Supplementary Data 9), ultimately indicating no pan-cancer association between pathway mutation and PM. Pan-pathway mutations in CRC HM, UCEC HM, and CSCC likely reflected their high TMB (Supplementary Fig. 3b). Stringent analysis (PM samples > 4, PM ratio > 0.05, effect size > 0.2 and FDR < 0.05) identified 6 significant subtype-pathway associations from 500 combinations (Fig. 5a, Supplementary Data 10). They were RTK-RAS (effect size = 0.71 and FDR = 0.001) and TGF-Beta (effect size = 0.73 and FDR = 0.010) in CRC MSS, TP53 in LUAD (effect size = 0.6 and FDR = 0.002), PRAD (effect size = 0.5 and FDR = 0.004), UCEC ENDO (effect size = 0.47 and FDR = 0.034), Hippo in UCEC HM (effect size = 2.78 and FDR = 0.022) (Fig. 5a). In line with previous findings that BRAF mutation and CMS4 subtype are risk factors for PM^21^, our results similarly implicate mutations in the RTK-RAS and TGF-Beta pathways in promoting PM occurrence in CRC (Fig. 5a). To corroborate these findings, we sought to replicate them in the CHORD cohort (*n* = 11,240). This analysis validated that mutations in the RTK-RAS (effect size = 0.74; FDR = 0.034) and TGF-Beta (effect size = 0.67; FDR < 0.001) pathways were associated with PM in colorectal cancer (Fig. 5b, Supplementary Data 11). Moreover, we identified significant associations with two additional pathways: WNT (effect size = 2.01; FDR < 0.001) and NOTCH (effect size = 1.46; FDR = 0.001). A retrospective analysis of the MetTropism dataset revealed that mutations in the WNT and NOTCH pathways also showed positive, albeit non-significant, trends (Supplementary Data 11). Thus, our findings on the association between pathway mutations and PM occurrence were successfully validated and expanded. To further characterize the mutational profile of the TGF-Beta pathway in MSS colorectal cancer, we analyzed its seven constituent genes across 3186 patients. Mutations in this pathway were present in 691 patients (21.69%; Supplementary Fig 3c). Among the five genes found to be mutated, SMAD4 was the most frequently altered (14% of patients), followed by SMAD3 (4%), SMAD2 (4%), TGFBR2 (2%), and TGFBR1 (1%) (Supplementary Fig 3c). These findings demonstrate that mutations in specific pathways are associated with PM.

### Mutational signature of PM

**Fig. 6:**
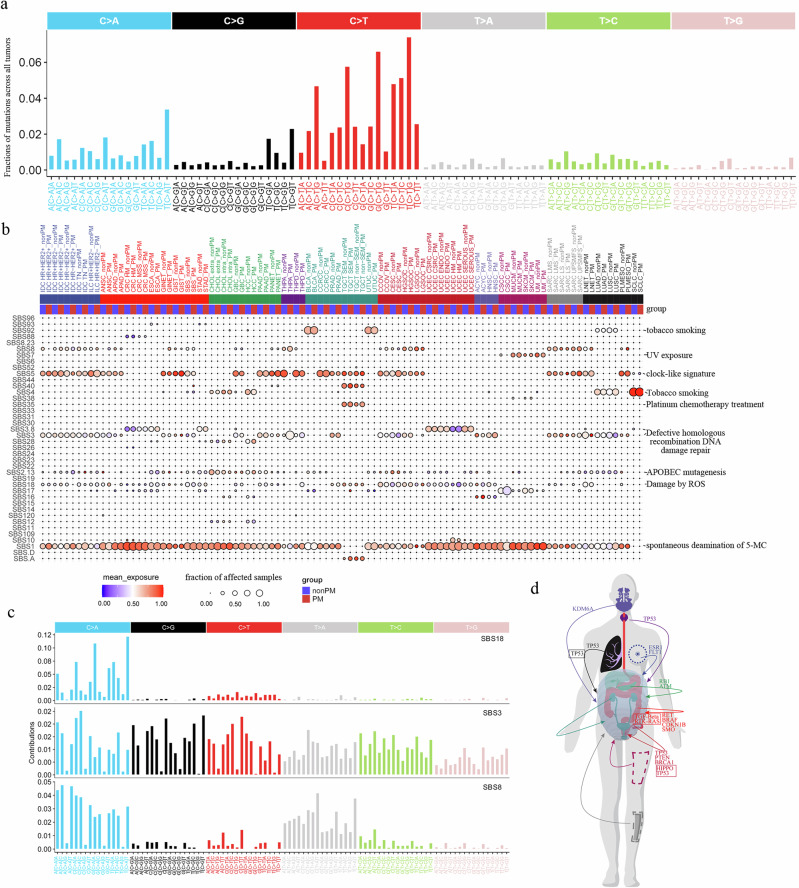
Mutational signatures of PM. **a** Distribution of 96 trinucleotide mutation types across the tumor cohort. Stacked bars represent the proportion of each substitution class (C > A, C > G, C > T, T > A, T > C, T > G) within its sequence context. **b** Comparison of mutational signature exposures (SBS1–SBS109) between peritoneal metastasis (PM) and non-PM samples across 50 cancer subtypes. Heatmap and scatter plots visualize signature activity differences. **c** Mutational characteristics of DNA damage repair-associated signatures SBS3, SBS8, and SBS18, highlighting their respective mutational profiles and contextual patterns. **d** Anatomical pathway mapping in peritoneal metastasis. Schematic illustration projects key genes and signaling pathways onto a human anatomical diagram to visualize their site-specific roles in PM dissemination.

Multiple mutational processes shape cancer genomes, each generating distinct mutational signatures. Characterizing peritoneal metastasis (PM) signatures could reveal key biological processes in PM development. We first visualized the distribution of 96 single-base substitution (SBS) classes (C > A, C > G, C > T, T > A, T > C, T > G with flanking context; COSMIC framework) across all patients (Fig. 6a) and PM/non-PM cohorts (Supplementary Fig 4). Using Deepsig—a deep-learning signature caller pretrained on whole-genome/MSK-IMPACT matched data—we cataloged 41 SBS signatures across 24,746 patients (Supplementary Data 12). Comparative analysis revealed significant dysregulation of SBS18, SBS3, and SBS8 in PM (Fig. 6b). SBS18 (ROS-induced DNA damage; C > A dominance) was enriched in PM of ANSC, APAD, GINET, STAD, PAAD, THPD, PRAD, CESC, and LGSOC. This is biologically significant as ROS drives metastasis through metabolic stress. SBS3 (homologous recombination deficiency; balanced substitutions) showed enrichment in GIST, SBS, PANET, and THPA PM. SBS8 (unknown etiology; C > A/T > A dominance) was elevated in PM from ILC HR + HER2- breast cancer, ANSC, GINET, THPA, CCOV, LNET, and PLMESO (Fig. 6b, c).

Taken together, we concluded genes and pathways whose mutation would lead to PM formation from 10 organ systems (Fig. 6d).

## Discussion

Peritoneal metastasis represents one of the most clinically challenging forms of cancer dissemination, characterized by limited treatment options and extremely poor prognosis. While predominantly observed in high-grade serous ovarian carcinoma (HGSOC), low-grade serous ovarian carcinoma (LGSOC), stomach adenocarcinoma (STAD), colorectal cancer (CRC), appendiceal adenocarcinoma (APAD), and pancreatic adenocarcinoma (PAAD), its relative rarity across other malignancies has resulted in insufficient mechanistic understanding. Leveraging the MSK-MetTropism cohort^22^—the largest metastatic cancer database—we systematically elucidate pan-cancer clinicopathological and genomic features of PM.

Overall, peritoneal metastasis (PM) occurred in 29% of metastatic patients on average, with incidence ranging from 1% in thyroid papillary carcinoma (THPA) to 92% in high-grade serous ovarian carcinoma (HGSOC). This reflects established organ tropism patterns^23^, as primary tumors originating from digestive and gynecologic systems demonstrated significantly higher PM frequency. While anatomical proximity and shared circulatory networks with the peritoneum may contribute, molecular drivers appear critical. Even within the same organ system, PM incidence varied substantially—evidenced by 29% occurrence in hormone receptor-positive HER2-negative invasive lobular breast carcinoma (ILC HR + HER2−) versus 11% in invasive ductal carcinoma (IDC HR + HER2−), and by 40.7% in microsatellite-stable colorectal cancer (CRC MSS) versus 26.3% in hypermutated CRC (CRC HM). These differentials strongly implicate subtype-specific molecular circuitry in PM pathogenesis.

Cytoreductive surgery (CRS) combined with hyperthermic intraperitoneal chemotherapy (HIPEC) represents the standard treatment for peritoneal metastasis (PM) regardless of primary tumor origin^24^. While significantly improving survival outcomes, this approach remains limited by high recurrence rates^25^. The somatic mutations enriched in PM may offer promising therapeutic targets. Although genomic profiling of PM has been reported in high-incidence malignancies, including gastric cancer (GC)^9^, colorectal cancer (CRC)^26^, pancreatic cancer^27^, high-grade serous ovarian carcinoma (HGSOC)^28^, and low-grade serous ovarian carcinoma (LGSOC)^29^, key mechanistic questions persist. In gastric cancer, the role of genomic alterations in PM development remains controversial: TCGA analyses revealed limited oncogenic pathway alterations in PM^30^, while Tanaka et al. reported enrichment of CDH1, SOX9, and EGFR in gastric PM versus primary tumors^31^. Our study identifies RET mutation as a potential driver of peritoneal metastasis (PM) in gastric cancer. Notably, all 7 RET-mutated GC patients (7/296, 2.4%) developed PM, and we further demonstrated significant upregulation of the RET signaling pathway in two independent GC PM cohorts. Moreover, the suppression of PM formation by pralsetinib in preclinical models provides functional validation of this finding. In colorectal cancer, Lenos et al. demonstrated KRAS/BRAF enrichment in PM^10^ which we confirm while additionally identifying SMO and RNF43 mutations in CRC MSS using our larger cohort (*n* = 3186). Paradoxically, despite KRAS’s presumed role in pancreatic cancer PM^32^, our analysis of 1970 pancreatic cases revealed no significantly enriched PM-associated genes, suggesting non-genomic drivers. Similarly, in HGSOC (96% TP53-mutated), no enriched mutations were observed, consistent with prior reports^33^. The limited LGSOC cohort (*n* = 70) precluded PM gene identification. Importantly, we discovered pan-cancer PM-associated mutation of ESR1 previously linked to metastasis, suggesting conserved molecular determinants across tumor types that could enable early detection and targeted therapies.

We further identified pathway-level alterations enriched in PM. While pathway-level mutations were not significantly associated with peritoneal metastasis (PM) occurrence in the pan-cancer analysis, we observed subtype-specific enrichment patterns. Specifically, TP53 pathway mutations were enriched in LUAD, PRAD, and UCEC endometrial subtypes. In colorectal cancer (CRC) microsatellite-stable (MSS) subtypes, RTK-RAS and TGF-Beta pathway mutations were prominent—a finding validated in the independent CHORD cohort. These results align with previous reports identifying BRAF mutation and CMS4 subtype (characterized by affected TGF-Beta pathway) as established risk factors for PM in CRC.

This study has several limitations that should be considered. (1) The analytical scope was largely confined to a single cohort, owing to the relatively low incidence of peritoneal metastasis (PM) among metastatic sites and the lack of larger publicly available datasets that systematically integrate PM status with genomic profiling. Although the CHORD cohort was used to validate key gene- and pathway-level findings, it also relies on targeted sequencing from a similar experimental platform, which may limit the independence of the validation. Additionally, since targeted sequencing covers only a small fraction of the genome, the genomic information available in this study was inherently restricted. (2) Although the genomic dataset included samples from primary tumors and common metastatic sites (e.g., liver and lymph nodes), PM-specific samples accounted for only approximately 650 cases. This limited sample size may introduce confounding effects related to tissue of origin when interpreting PM-associated genomic alterations. (3) Functional validation was conducted only in gastric cancer models. The majority of other findings, particularly those involving additional cancer types, have not yet been substantiated through gene-oriented functional studies, highlighting an important direction for future investigation. (4) Classical mutational signatures are defined by whole-genome sequencing (WGS) data, and whether they can be reliably predicted from targeted sequencing data remains a subject of debate. Although DeepSig was developed to infer signatures from such data, its application has not seen widespread adoption. Consequently, future studies directly profiling PM tissues are warranted.

Collectively, we provide comprehensive pan-cancer analyses of PM clinicopathological features and genomic alterations. These results establish a foundational resource for advancing our understanding of peritoneal dissemination mechanisms.

## Methods

### Datasets

The MSK-MetTropism data was downloaded from Cbioportal at https://www.cbioportal.org/study/summary?id=msk_met_2021. CHORD dataset was from Cbioportal at https://www.cbioportal.org/study/summary?id=msk_chord_2024. The metastatic breast cancer dataset, which includes both mutational and transcriptomic (RNA-seq) profiles, was sourced from cBioPortal https://www.cbioportal.org/study/summary?id=brca_mbcproject_2022. GSE190609, GSE183202, GSE289037 and GSE66229 were from GEO at https://www.ncbi.nlm.nih.gov/geo/. TCGA-COAD (GDC dataset) was from Cbioportal.

### Visualization

Summarized data were visualized by the R package *ggpubr* unless otherwise referred. Specifically, Pie figures were visualized by ggnestedpie with parameters indicated in the uploaded code. A stacked barplot was accomplished by ggbarplot. A scattered dot plot was performed by ggscatter. The Kaplan–Meier survival curve was drawn by ggplot2. The heatmap was visualized by *heatmap*. Volcanoplot was done by *EnhancedVolcano*. Forest plot was accomplished with R package *forestploter*.

### Odds ratios for peritoneal metastasis across cancer subtypes

To quantify the risk of peritoneal metastasis (PM) occurrence for each cancer subtype in the pan-cancer cohort, we fitted a multivariable logistic regression model adjusted for the following covariates: cancer subtype, gene panel, fraction of genome altered (FGA), microsatellite instability (MSI) status, tumor mutational burden (TMB), sex, age, and race. The resulting odds ratios (ORs) and their 95% confidence intervals (CIs) are reported for each subtype. *P*-values were adjusted for multiple testing using the Benjamini-Hochberg method.

### Survival analysis

Survival analyses were performed using Cox proportional hazards models implemented with the R packages *survival* and *survminer*. For cancer subtype-specific analyses, we fitted a multivariable Cox regression model that included the following covariates: metastatic sites, gene panel, sample type, fraction of genome altered (FGA), microsatellite instability (MSI), tumor mutational burden (TMB), sex, age, race, and peritoneal metastasis (PM) status. The resulting hazard ratios (HRs) with 95% confidence intervals were visualized using the *forestploter* and *ggplot2* packages. *P*-values from the 39 cancer subtype analyses were adjusted for multiple testing using the False Discovery Rate (FDR) method. Detailed analytical code has been made publicly available.

### Pan-Cancer analysis of peritoneal metastasis risk Genes

To identify genes associated with the risk of peritoneal metastasis (PM) across cancer types, we constructed a multivariable logistic regression model. The model included 489 candidate genes, with adjustment for cancer subtype, sex, and gene panel version. Resulting *p*-values were adjusted for multiple testing using the Benjamini–Hochberg false discovery rate (FDR) method. Significant associations were visualized using a volcano plot, and full results are available in Supplementary Data 4. The complete analytical workflow is provided in the publicly available code. For genes harboring hotspot mutations, we applied an identical analytical pipeline but restricted the gene set to 232 candidates with documented hotspot mutations obtained from the Cancer Hotspots database (https://www.cancerhotspots.org/, PMID: 29247016). The hotspot mutation profile of ESR1 was visualized using the *lollipopPlot* function from the R package *maftools*. For the identification of risk genes through the integration of mutations and copy number alterations (CNA), we first constructed a unified alteration matrix by labeling genes as “changed” if affected by either type of event. This matrix was then analyzed using a multivariable logistic regression model adjusted for sex, gene panel, and cancer subtype. Resulting *p*-values were adjusted via the Benjamini–Hochberg FDR procedure, and significant associations were visualized in a volcano plot.

### Cancer subtype analysis of peritoneal metastasis risk genes

Somatic mutation data were analyzed using the R package *maftools*. The *oncoplot* function was employed to visualize the mutational landscape of frequently altered or selected genes across the entire cohort or within specified cancer types. To identify genes significantly enriched or depleted in each cancer type, we performed comparative analyses using the *mafCompare* function. Resulting *p*-values were adjusted for multiple testing using the Benjamini–Hochberg false discovery rate (FDR) method. All analyses can be reproduced by executing the provided code.

### Deconvolution of transcription factor and pathway activity scores from transcriptomic data

Bulk RNA-seq data were obtained from the Gene Expression Omnibus (GEO) as indicated previously and preprocessed into a gene expression matrix with samples as columns and genes as rows. Transcription factor (TF) activities were decoupled using weighted TF–gene interaction pairs from the *DoRothEA* database, followed by decomposition with the R package *decoupleR*. Resulting activity scores were computed as the consensus of four distinct statistical methods. For RET signaling pathway assessment, signature genes were acquired from MSigDB, and scoring was performed using an algorithm adapted from the *AddModuleScore* function in *Seurat*. Complete reproducibility of all analyses is ensured through the uploaded code.

### Evaluation of RET inhibitor efficacy against gastric cancer peritoneal metastasis

The RET inhibitor pralsetinib, which is clinically used, was obtained from MedChemExpress (Cat. No. HY-112301). The mouse gastric cancer cell line MFC was acquired from the National Collection of Authenticated Cell Cultures of Chinese Academy of Sciences (Cat. No. TCM23). MFC cells were maintained in DMEM (Gibco) supplemented with 10% fetal bovine serum and 1% penicillin/streptomycin (Sangon Biotech), and cultured at 37 °C in a humidified atmosphere containing 5% CO₂. To establish the peritoneal metastasis model, cells were harvested at 70–90% confluence. Each mouse received an intraperitoneal injection of 1 × 10⁶ cells suspended in 100 µL of PBS, with six mice per experimental group. Pralsetinib was dissolved in DMSO and further diluted in PBS for administration. Mice were treated via intraperitoneal injection with 100 µL of pralsetinib at a dose of 10 mg/kg every three days. Two weeks after model establishment, all mice were euthanized and subjected to macroscopic imaging for evaluation of tumor burden.

### Evaluating PM on tumor progression after therapy

We obtained patient treatment and progression information from the CHORD dataset. The analysis included 3078 patients (842 with PM) from the MetTropism cohort and 1770 patients (415 with PM) from the CHORD cohort, all of whom had available therapy records (identified by patient number and treatment start date). The final consolidated dataset comprised 5752 patient–therapy records, with some patients contributing multiple lines of therapy. To assess the influence of PM status on treatment outcome, we fitted a multivariable logistic regression model adjusted for therapy modalities and cancer subtype. For the colorectal cancer (CRC) subgroup, we further evaluated the association between PM status and outcomes of specific therapies by incorporating interaction terms into a logistic regression model. All models were implemented using the R package *glmnet*.

### Pathway calculation

Pathway-level alteration burden was assessed using the pathways function on the MAF object, which summarized mutation events across a defined set of 10 pathways (Sanchez-Vega et al., 10.1016/j.cell.2018.03.035) for all samples in the cohort. For mutation rate of pathway genes, size effect was calculated as Hedge’s g in R package *effectsize*. To identify pathways enriched or depleted in peritoneal metastasis (PM), we applied a consistent pipeline to each cancer subtype. For each pathway, a 2 × 2 contingency table was constructed and subjected to Fisher’s exact test. The effect size was estimated as an odds ratio [OR = (a × d)/(b × c)], and *p*-values were adjusted for multiple testing using the Benjamini–Hochberg false discovery rate (FDR) method. Results were integrated across all subtypes and visualized as implemented in the accompanying code.

### Mutational signature

The mutational signature was predicted by the *DeepSig* R package. We followed the tutorial to predict mutational signatures for each cancer type. Briefly, SBS-96 matrix was generated by R package *sigminer* with function sig_tally using MAF object containing all mutational data. Deep-learning model used was v0.99. Prediction was accomplished by the function DL.call. We got a total of 41 SBS signatures for 24746 patients comprising all 50 cancer subtypes (Supplementary Data 12).

## Supplementary information

Supplementary information

Supplementary Data 1

Supplementary Data 2

Supplementary Data 3

Supplementary Data 4

Supplementary Data 5

Supplementary Data 6

Supplementary Data 7

Supplementary Data 8

Supplementary Data 9

Supplementary Data 10

Supplementary Data 11

1-Supplementary Data 12

2-Supplementary Data 12

supplemetnal Tables

## Data Availability

Datasets are provided in the methods part. All processed data can be requested from the corresponding author at chentianwei@sibs.ac.cn.

## Code availability

The codes generated during this study are available at Figshare: 10.6084/m9.figshare.29665145.
