# Supplementary material for: Pan-cancer clinicopathological and genomic characteristics of peritoneal metastasis
Source: NPJ Precis Oncol. 2025 Dec 13;10:34. doi: 10.1038/s41698-025-01227-7 (PMC12820284; doi:10.1038/s41698-025-01227-7)
Supplement: Supplementary file 1 — Supplementary information [file 41698_2025_1227_MOESM1_ESM.pdf]

Pan-cancer clinicopathological and genomic characteristics of peritoneal metastasis

By Chen et al.

Supplementary Files

Supplementary Figures

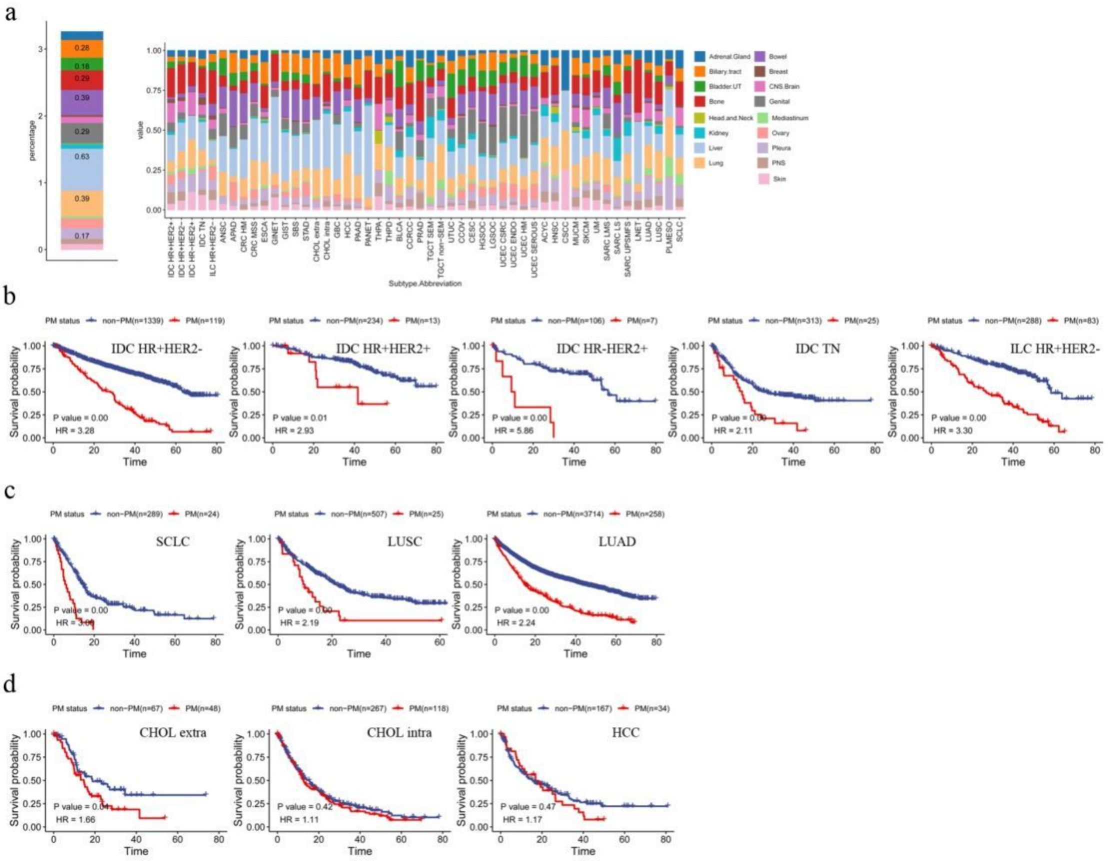

Supplementary Figure 1. The impact of PM on overall survival across diverse cancer subtypes. (a) Distribution of concurrent metastatic sites in patients with peritoneal metastasis (PM). Stacked bar plots show the profile for the entire MetTropism cohort (left) and across 50 cancer subtypes (right). (b-d) Kaplan-Meier survival curve of breast cancer subtypes (b, IDC HR+HER2-, IDC HR+HER2+, IDC HR-HER2+, IDC TN, ILC HR+HER2-), demonstrating significantly worse survival in PM cases compared to non-PM controls ($P < 0.001$ for all subtypes, HR ranging from 2.11 to 3.28), and lung cancers (c, SCLC, LUSC LUAD) exhibiting rapid decline ($P < 0.001$), and gastrointestinal cancers (d, CHOL extra, CHOL intra, HCC).

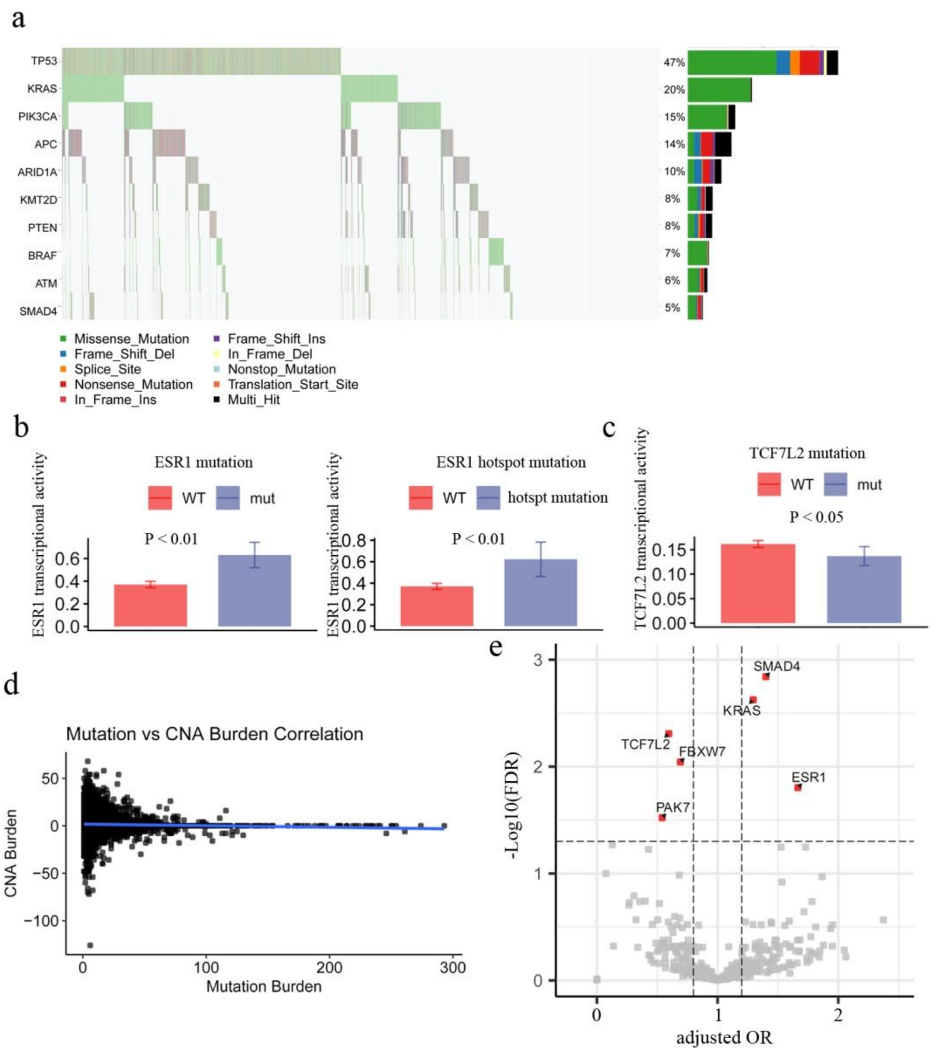

Supplementary Figure 2. PM mutation patterns across diverse cancer types. (a) Waterfall plot illustrating the distribution of hotspot mutation in non-PM patients. (b) ESR1 transcriptional activity in a metastatic breast cancer cohort. Bar plot shows deconvoluted transcriptional activity levels from bulk RNA-seq data, comparing wild-type versus mutant tumors (left) and wild-type versus tumors with hotspot mutations (right). (c) TCF7L2 transcriptional activity in TCGA-COAD (GDC) cohort. Bar plot shows deconvoluted transcriptional activity levels from bulk RNA-seq data, comparing wild-type versus mutant tumors (left). (d) Correlation between gene mutation and respective CAN. (e) Genetic alterations associated with peritoneal metastasis. Volcano plot shows significantly enriched and depleted genes in PM compared to non-PM samples

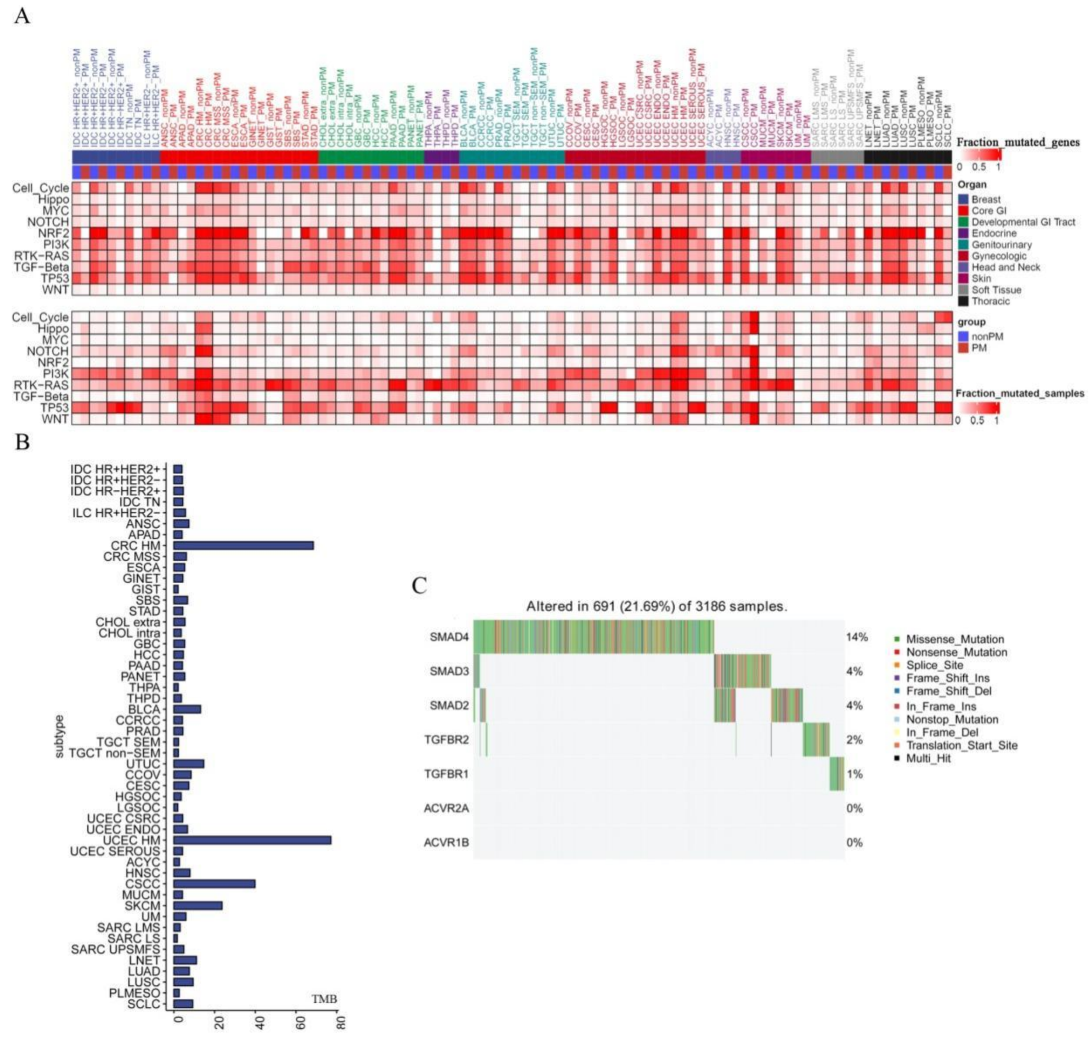

Supplementary Figure 3. Pathway mutation across cancer subtypes. (a) Heatmap comparing the fraction of mutated genes in 10 key pathways between metastatic (PM) and non-metastatic (non-PM) samples across 10 organ systems. (b) Bar plot showing tumor mutational burden (TMB) distributions for 28 cancer subtypes, with blue bars representing median TMB values. (c) Mutational landscape of the TGF-Beta pathway genes in the colorectal cancer (CRC) subcohort of the MetTropism study.

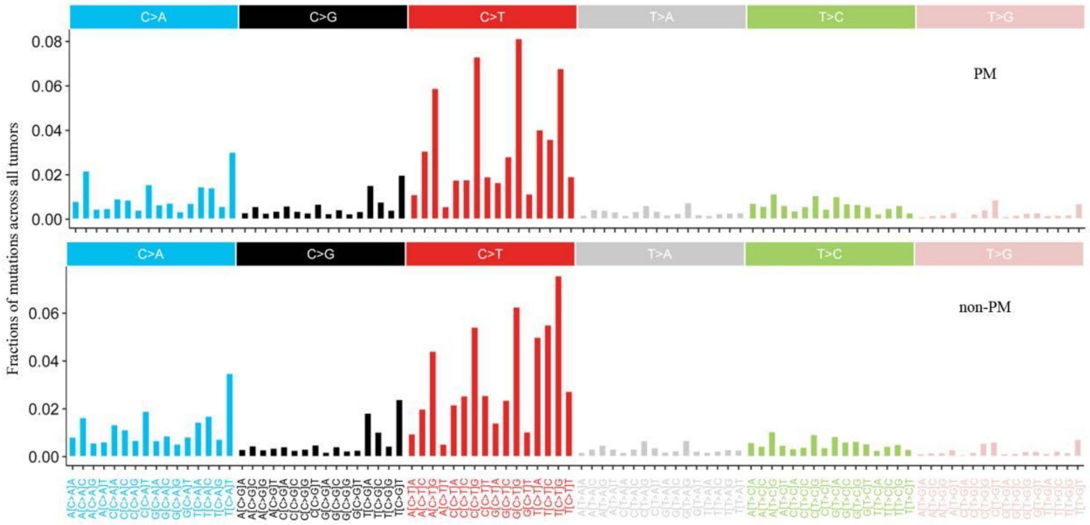

Supplementary Figure 4. Mutation signature of PM and non-PM. Bar plot showing catalogue of 96 signatures in PM (upper panel) patients and non-PM patients (lower panel).

Supplementary Data

Supplementary Data 1. Dissemination pattern of PM patients across 50 cancer subtypes

Supplementary Data 2. Result of multivariable logistic regression evaluating odd ratios of PM occurrence across 50 cancer subtypes.

Supplementary Data 3. Association between cancer subtypes and overall survival: a multivariable Cox regression analysis.

Supplementary Data 4. Risk genes of PM in a pan-cancer multivariable logistic regression model.

Supplementary Data 5. Risk genes carrying hotspot mutations in a pan-cancer multivariable logistic regression model.

Supplementary Data 6. Risk genes of PM in a pan-cancer multivariable logistic regression model incorporating CNA.

Supplementary Data 7. Risk genes of PM for each cancer subtypes.

Supplementary Data 8. gene list of 10 pathways.

Supplementary Data 9. Mutated pathway gene ratio and sample ratio comparison between PM and non-PM groups.

Supplementary Data 10. Differential enrichment of mutated pathways in PM versus non-PM samples.

Supplementary Data 11. Differential enrichment of mutated pathways in PM versus non-PM samples of CHORD set.

Supplementary Data 12. DeepSig result of each cancer subtype.
